# 1057. Development of a Flow Cytometry-Based Micro-Neutralisation Assay to Evaluate Humoral Immunity Against SARS-CoV-2 Variants of Concern in Vaccine Trials

**DOI:** 10.1093/ofid/ofac492.898

**Published:** 2022-12-15

**Authors:** Sophie R O’Reilly, Grace Kenny, Tamara Alrawahneh, Nathan Francois, Matthew Angeliadis, Valentin de Masson d’Autume, Alejandro Garcia-Leon, Eoin Feeney, Obada Yousif, Aoife Cotter, Eoghan de Barra, Mary Horgan, Patrick Mallon, M B BCh, Virginie Gautier

**Affiliations:** Centre for Experimental Pathogen Host Research (CEPHR), University College Dublin, Dublin, Dublin, Ireland; Centre for Experimental Pathogen Host Research (CEPHR), University College Dublin, Belfield, Dublin 4, Ireland; Department of Infectious Diseases, St Vincent’s University Hospital, Elm Park, Dublin 4, Ireland, Dublin, Dublin, Ireland; Centre for Experimental Pathogen Host Research (CEPHR), University College Dublin, Belfield, Dublin 4, Ireland, Dublin, Dublin, Ireland; Centre for Experimental Pathogen Host Research (CEPHR), University College Dublin, Belfield, Dublin 4, Ireland, Dublin, Dublin, Ireland; Centre for Experimental Pathogen Host Research (CEPHR), University College Dublin, Belfield, Dublin 4, Ireland, Dublin, Dublin, Ireland; Centre for Experimental Pathogen Host Research (CEPHR), University College Dublin, Belfield, Dublin 4, Ireland, Dublin, Dublin, Ireland; Centre for Experimental Pathogen Host Research (CEPHR), University College Dublin, Belfield, Dublin 4, Ireland, Dublin, Dublin, Ireland; Department of St Vincent's University Hospital and Centre for Experimental Pathogen Host Research University College Dublin, Belfield, Dublin, Ireland; 4Endocrinology Department, Wexford General Hospital, Carricklawn, Wexford, Ireland, Wexford, Wexford, Ireland; Centre for Experimental Pathogen Host Research (CEPHR), University College Dublin, Belfield, Dublin 4, Ireland; Department of Infectious Diseases, Mater Misericordiae University Hospital, Eccles St, Dublin 7, Ireland, Dublin, Dublin, Ireland; 6Department of Infectious Diseases, Beaumont Hospital, Beaumont, Dublin 9, Ireland; Department of International Health and Tropical Medicine, Royal College of Surgeons in Ireland, Dublin, Ireland, Dublin, Dublin, Ireland; Department of Infectious Diseases, Cork University Hospital, Wilton, Co Cork, Ireland, Cork, Cork, Ireland; University College Dublin, Dublin, Dublin, Ireland; Centre for Experimental Pathogen Host Research (CEPHR), University College Dublin, Belfield, Dublin 4, Ireland, Dublin, Dublin, Ireland

## Abstract

**Background:**

Quantifying neutralising capacity of circulating SARS-COV-2 antibodies is critical in evaluating protective humoral immune responses generated post-infection/post-vaccination.

Here we describe a novel medium-throughput flow cytometry based micro-neutralisation assay to evaluate Neutralising Antibody (NAb) responses against live SARS-CoV-2 Wild Type (D641G) and Variants of Concern (VoC) in convalescent/vaccinated populations.

**Methods:**

Micro-Neutralisation assay (Micro-NT) was performed in 96-well plates using clinical isolate 2019-nCoV/Italy-INMI1, D641G (SARS-CoV-2/human/IRL/AIIDV1446/2020) and/or VOCs Beta (SARS-CoV-2/human/IRL/AIIDV1752/2021) and Omicron (SARS-Cov-2/human/IRL/AIIDV2326/2021). Plasma samples (All Ireland Infectious Diseases (AIID) Cohort) were serially diluted (8 points, half-log) from 1/20 and pre-incubated with SARS-CoV-2 (1h, 37°C). Virus-plasma mixture were added onto VERO E6/VERO-E6 TMPRSS2 cells for 18h. Percentage infected cells was analysed by automated flow cytometry following trypsinisation, fixation and SARS-CoV-2 Nucleoprotein intracellular staining. Half-maximal Neutralisation Titres (NT50) was determined using four-parameter logistic regression. Our assay was compared to Plaque Reduction Neutralisation Test (PRNT) and validated against WHO anti-SARS-CoV-2 Immunoglobulin Standards.

**Results:**

Using WHO Standards with low, medium or high anti-SARS-CoV-2 IgG, both Micro-NT and PRNT achieved comparable NT50 values (Table 1). Micro-NT was found to be highly reproducible (inter-assay CV of 11.39%). Screening 190 convalescent samples and 11 COVID-19 naive controls (AIID cohort) we achieved an assay sensitivity of 90% and specificity of 81%. We demonstrated that Micro-NT has broad dynamic range differentiating NT50s < 1/20 to > 1/5000 (Figure 1). We could also characterise immune-escape VoC, observing up to 10-fold reduction in NT50 against Beta (Figure 2).

**Table 1:**
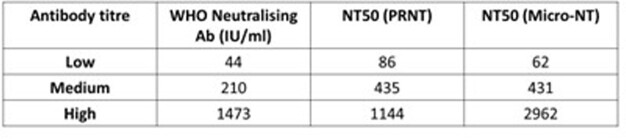
NT50s of Low, Medium and High Titre Anti-SARS-CoV-2 IgG Standards measured against Live SARS-CoV-2 using PRNT and Micro-NT

Neutralising Capacity of low, medium and high-titre anti-SARS-CoV-2 IgG (WHO, International Standards) against live SARS-CoV-2 (2019-nCoV/Italy-INMI1) measured using PRNT and Micro-NT Assays on Vero E6 cells, as well as the potency of NAbs in each sample in International Units (IU/ml) as determined by the WHO.

**Figure 1:**
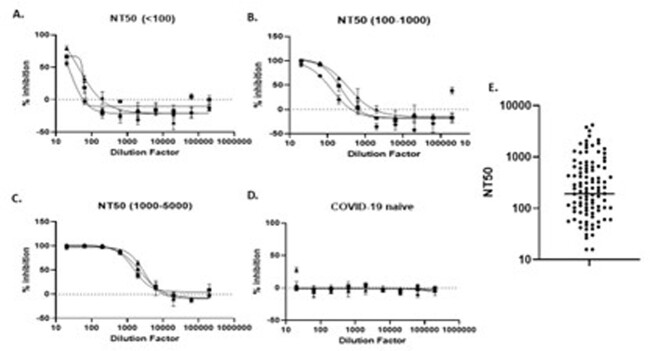
Dynamic Range of Micro-NT

Micro-NT has a broad Dynamic Range, distinguishing low (A), medium (B) and high (C) neutralising plasma samples against live SARS-CoV-2 (2019-nCoV/Italy-INMI1) from a cohort of COVID-19 convalescent individuals (AIID cohort), as well as negative samples from COVID-19 naïve samples (D). Graphs show 3 representative samples of each NT50 range. (E) shows the population distribution of 190 Convalescent plasma samples as measured by Micro-NT on Vero E6 cells.

**Figure 2:**
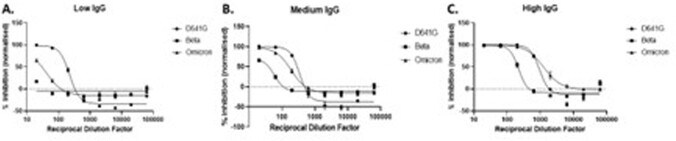
Reduced Neutralisation Capacities measured against SARS-CoV-2 VoC using Micro-NT

Low (A), Medium (B) and High (C) anti-SARS-CoV-2 IgG (WHO Standards) show different neutralising capacities against WT (D614G) SARS-CoV-2 and variants Beta and Omicron, measured using Micro-NT on Vero-E6-TMPRSS2 cells.

**Conclusion:**

Our flow-cytometry-based Micro-NT is a robust and reliable assay to quantify NAb titres, an important evaluation endpoint in clinical trials. It has higher throughput (96 well format versus 12 well) and reduced infection time (18h vs 48-96h) compared to the gold standard PRNT.

**Disclosures:**

**All Authors**: No reported disclosures.

